# The Effect of hOGG1 Ser326Cys Polymorphism on Cancer Risk: Evidence from a Meta-Analysis

**DOI:** 10.1371/journal.pone.0027545

**Published:** 2011-11-17

**Authors:** Bingbing Wei, You Zhou, Zhuoqun Xu, Bo Xi, Huan Cheng, Jun Ruan, Ming Zhu, Qiang Hu, Qiang Wang, Zhirong Wang, Zhiqiang Yan, Ke Jin, Deqi Zhou, Feng Xuan, Xing Huang, Jianfeng Shao, Peng Lu

**Affiliations:** 1 Department of Urology, Affiliated Wuxi People's Hospital of Nanjing Medical University, Wuxi, China; 2 Minerva Foundation Institute for Medical Research, Biomedicum 2U, Helsinki, Finland; 3 Department of Maternal and Child Health Care, School of Public Health, Shandong University, Jinan, China; 4 Department of Urology, First Affiliated Hospital of Nanjing Medical University, Nanjing, China; Florida International University, United States of America

## Abstract

**Background:**

Human oxoguanine glycosylase 1 (hOGG1) in base excision repair (BER) pathway plays a vital role in DNA repair. Numerous epidemiological studies have evaluated the association between hOGG1 Ser326Cys polymorphism and the risk of cancer. However, the results of these studies on the association remain conflicting. To derive a more precise estimation of the association, we conducted a meta-analysis.

**Methodology/Principal Findings:**

A comprehensive search was conducted to identify the eligible studies of hOGG1 Ser326Cys polymorphism and cancer risk. We used odds ratios (ORs) with 95% confidence intervals (CIs) to assess the strength of the association. We found that the hOGG1 Ser326Cys polymorphism was significantly associated with overall cancer risk (Cys/Cys vs. Ser/Ser: OR = 1.19, 95%CI = 1.09–1.30, *P*<0.001; Cys/Cys vs. Cys/Ser+Ser/Ser: OR = 1.16, 95%CI = 1.08–1.26, *P*<0.001). Moreover, in subgroup analyses by cancer types, the stronger significant association between hOGG1 Ser326Cys polymorphism and lung cancer risk was found (Cys/Cys vs. Ser/Ser: OR = 1.29, 95%CI = 1.16–1.44, *P*<0.001; Cys/Cys vs. Cys/Ser+Ser/Ser: OR = 1.22, 95%CI = 1.12–1.33, *P*<0.001). The significant effects of hOGG1 Ser326Cys polymorphism on colorectal, breast, bladder, prostate, esophageal, and gastric cancer were not detected. In addition, in subgroup analyses by ethnicities, we found that the hOGG1 Ser326Cys polymorphism was associated with overall cancer risk in Asians (Cys/Cys vs. Ser/Ser: OR = 1.21, 95%CI = 1.10–1.33, *P*<0.001).

**Conclusions:**

This meta-analysis showed that hOGG1 326Cys allele might be a low-penetrant risk factor for lung cancer.

## Introduction

DNA damage plays a vital role in carcinogenesis [1], which generally occurs through different mechanisms such as by-product of normal cellular metabolism or the result of exposure to biological and environmental mutagens. DNA damage, if it is not repaired, could lead to apoptosis or mutation, which may cause induction of carcinogenesis [1]. It is suggested that reactive oxygen species (ROS) could induce both base lesions and single strand breaks in DNA [1]. The 8-hydroxy-2-deoxyguanine (8-OH-dG) is a major form of DNA damage, which is produced by reactive free radicals.

The presence of 8-OH-dG in DNA is thought to be a major cause of G:C to T:A transversion, because 8-OH-dG could direct the incorporation of adenine as well as cytosine opposite the lesion [2]. Thus, 8-OH-dG is a highly mutagenic DNA lesion in vivo [3], [4] unless it is repaired prior to DNA replication. The DNA repair enzyme human oxoguanine glycosylase 1 (hOGG1) is a DNA glycosylase/AP lyase that has been indicated to play an important role in preventing carcinogenesis by repairing oxidative damage to DNA [5]. Specifically, glycosylase/AP lyase could efficiently catalyze the excision and removal of 8-OH-dG adducts. HOGG1 may play a vital role in maintaining genome integrity and preventing the development of cancer.

Genetic variations in hOGG1 gene are increasingly studied for an elevated cancer risk because of the critical roles in stabilizing genome integrity. The hOGG1 gene has codon 326 polymorphism (Ser326Cys, rs1052133), and Cys326 has lower ability to prevent mutagenesis by 8-OH-dG than Ser326 in human cells in vivo [5]. So far, there were so many reports about the association of hOGG1 Ser326Cys polymorphism with risk of different cancers, including breast [6]–[18], prostate [19]–[25], pancreatic [26], [27], bladder [28]–[34], gallbladder [35]–[38], gastric [39]–[49], colorectal [50]–[63], esophageal [64]–[68], lung [69]–[85], cervical cancers [86], [87], and so on [88]–[101].

One study showed that the hOGG1 Ser326Cys polymorphism was associated with an increased risk of colorectal cancer (odds ratio: 2.3; 95% confidence interval: 1.1–5.0), the risk being higher in younger individuals [60]. Canbay et al [63] found that hOGG1 Ser326Cys polymorphism might be associated with increased risk of colorectal cancer in a Turkish population. However, other studies [53], [54], [59] did not show the significant association between the Ser326Cys polymorphism and colorectal cancer. Numerous studies and systematic approaches examined the role of the Ser326Cys polymorphism in lung cancer susceptibility. One meta-analysis showed that the overall odds ratio of homozygote for the hOGG1 326Cys allele against those for the hOGG1 326Ser allele was 1.24 (95% confidence interval: 1.01–1.53), suggesting that the locus was involved in susceptibility to lung cancer [83]. In contrast, another meta-analysis reported no significant association [102]. Some studies [15], [16] indicated that the Ser326Cys polymorphism was not associated with breast cancer. However, Sangrajrang et al [11] found that Thai women with variant allele of hOGG1 were likely to have an increased susceptibility to breast cancer. In addition, Chen et al [24] found that hOGG1 Ser326Cys polymorphism was associated with prostate cancer risk whereas Nock et al [22] did not find the significant association in the total study population.

On the whole, the results about the association between hOGG1 Ser326Cys polymorphism and cancer risk were conflicting and inconclusive. To derive a more precise estimation of the association, we performed a meta-analysis.

## Materials and Methods

### Identification and eligibility of relevant studies

PubMed (1956 to 30 July 2011) and Embase (1947 to 30 July 2011) database search was performed using following search terms: “oxoguanine glycosylase 1, hOGG1 or OGG1”, “polymorphism or variant”, and “cancer, neoplasm or tumor”. Additional studies were identified by a hand search of the references of original studies. In case of the studies with the same or overlapping data, we selected the most recent ones with the largest number of subjects. Studies included in this meta-analysis should meet the following criteria: (a) evaluation the association of hOGG1 Ser326Cys polymorphism and cancer risk published in English language, (b) use a case-control design, (c) contain available genotype frequency, and (d) the distribution of genotypes in the controls was consistent with Hardy-Weinberg equilibrium (HWE).

### Data extraction

Two investigators independently extracted the data and reached a consensus on all the items. For each study, the following characteristics were collected: last name of first author, year of publication, country of origin, ethnicity, numbers of genotyped cases and controls. Different ethnic descents were categorized as Caucasians (at least 80% of Caucasians included), Asians, and Africans. If a study did not state the ethnic descendent or if it was not possible to separate participants according to such phenotype, the group reported was termed “mixed ethnicity”. In addition, if only one cancer type was included in a study in the meta-analysis, it was combined into the “mixed cancer” group. For study [49] including subjects of different ethnic groups, data were extracted separately for each ethnic group whenever possible. Because the studies [19], [31], [56], [87], [103] only provided the information of genotypes as “Cys/Cys+Cys/Ser” and Ser/Ser without data for other genotypes, we could only calculate the OR for the dominant genetic model.

### Statistical analysis

The strength of the association between hOGG1 Ser326Cys polymorphism and cancer risk was measured by odds ratios (ORs) with 95% confidence intervals (CIs). We first estimated the risks of the Cys/Cys and Ser/Cys genotypes on risk of cancer, compared with the wild-type Ser/Ser homozygote, then evaluated the risks of “Cys/Cys+Ser/Cys vs. Ser/Ser” and “Cys/Cys vs. Ser/Cys+Ser/Ser” on risk of cancer, assuming dominant and recessive effects of the variant Cys allele, respectively. Subgroup analysis was also performed based on different ethnicities, cancer types, age, and sex.

Heterogeneity was evaluated with a chi-square-based Q test among the studies (*P*<0.10 was considered significant) [104], [105]. When the heterogeneity was present, the random effects model was used to calculate the pooled OR [106], whereas the fixed effects model was used in its absence [107]. Sensitivity analysis was performed to assess the stability of the results.

For control group of each study, the allelic frequency was calculated, and the observed genotype frequencies of the hOGG1 Ser326Cys polymorphism were assessed for Hardy-Weinberg equilibrium (HWE) by using the Pearson chi-square test; *P*<0.05 was considered significant. Funnel plots and Egger's linear regression test were used to provide diagnosis of the potential publication bias [108].

All statistical tests for this meta-analysis were performed with STATA (version 10.0; Stata Corporation, College Station, TX) and SPSS for Windows (version 11.0; SPSS, Inc., Chicago, IL).

## Results

### Study characteristics

**Figure 1:**
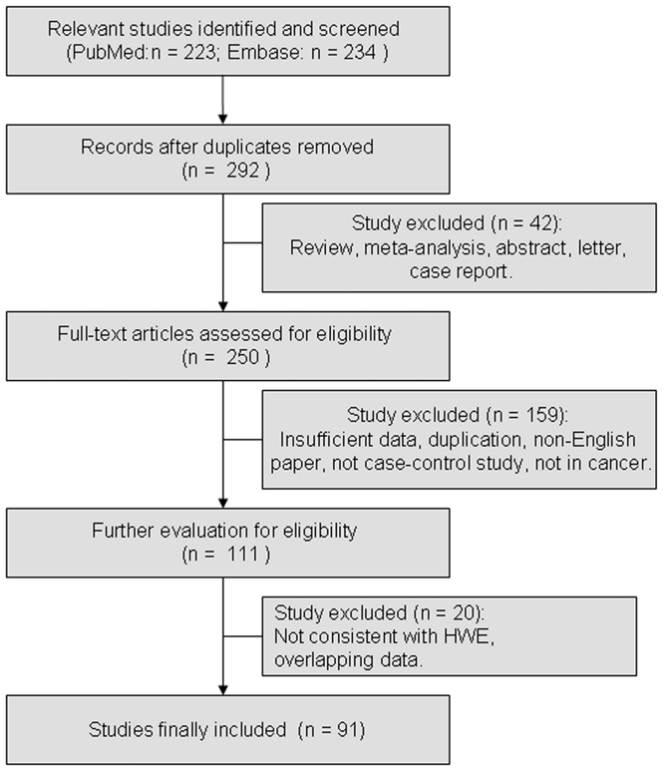
Flow chart of study selection based on the inclusion and exclusion criteria.

For cancer susceptibility related to hOGG1 Ser326Cys polymorphism, articles were retrieved based on the search criteria. Study selection process was shown in Figure 1. Among them, the distribution of genotypes in the controls was not consistent with HWE in 13 studies, which were excluded in the meta-analysis. 5 additional studies were excluded because of overlapping data. Finally, a total of 91 case-control studies involving 31,297 cancer cases and 39,033 controls were included in the meta-analysis. The characteristics of included studies were summarized in Table S1. There were 42 studies of Caucasian descendants and 35 studies of Asian descendants. Cancers were confirmed histologically or pathologically in most studies. There were 14 studies of colorectal cancer, 19 studies of lung cancer, 12 studies of breast cancer, 6 studies of bladder cancer, 4 studies of prostate cancer, 11 studies of gastric cancer, 5 studies of esophageal cancer, 6 studies of head and neck cancer, 2 studies of gallbladder cancer, and 2 studies of ALL. There were 57 studies, in which the data on age of cancer cases and controls were shown in detail. Among them, the age-matched control subjects were used in 42 studies, which were included in subgroup analyses by age. 19 studies, which specifically reported data according to gender, were eligible for subgroup analyses by sex. In addition, the distribution of genotypes in the controls was consistent with HWE in all studies (*P*>0.05).

### Quantitative synthesis

The 326Cys allele frequencies in controls of different ethnicities were calculated. The frequency of the 326Cys allele was 47.07% (95%CI = 43.39–50.75%) among Asian controls, which was significantly higher than that of Caucasian controls (23.62%; 95%CI = 20.43–26.81%, *P*<0.001; Figure S1).

**Figure 2:**
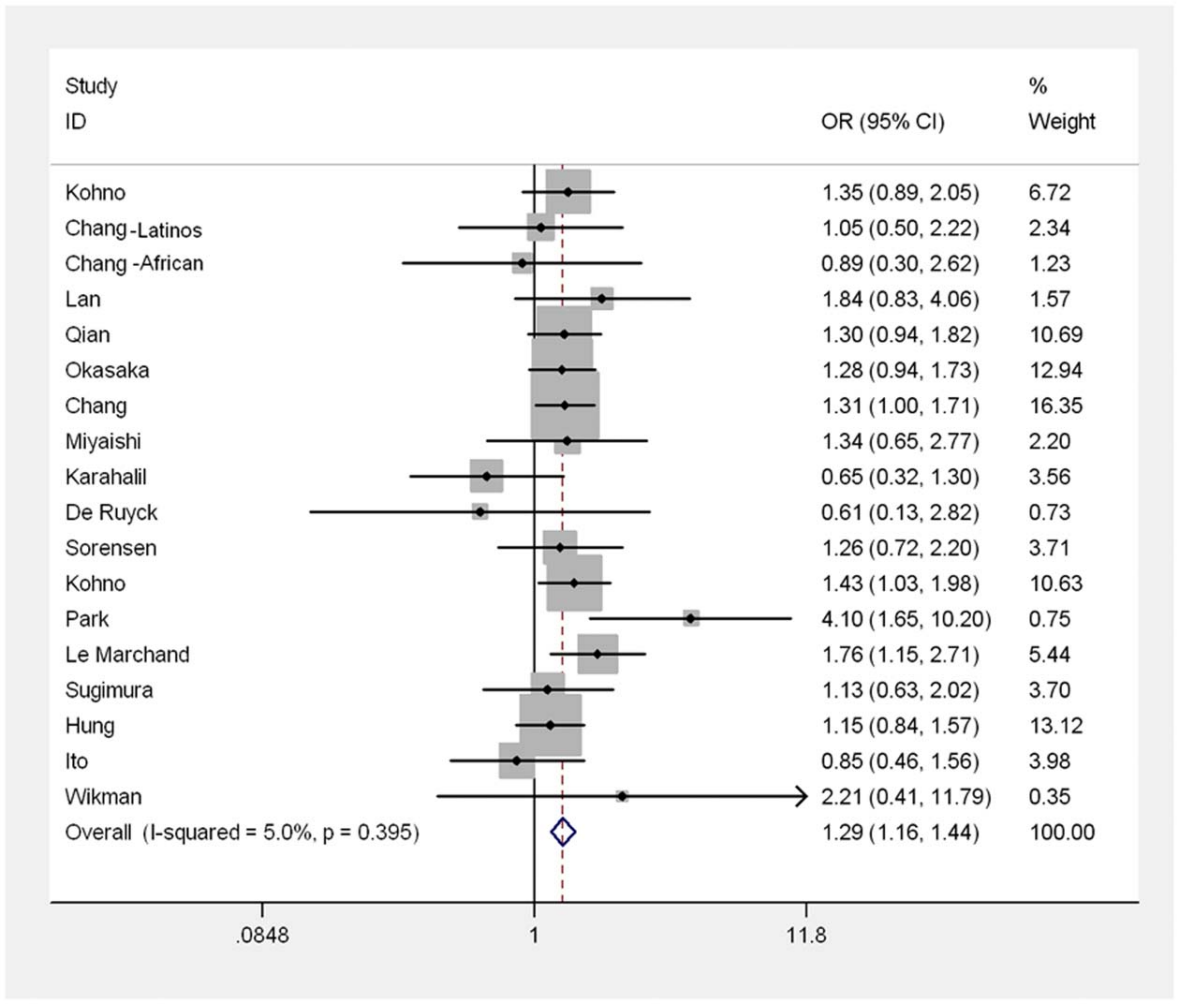
Forest plot of lung cancer risk associated with hOGG1 Ser326Cys polymorphism (for Cys/Cys vs. Ser/Ser). The squares and horizontal lines correspond to the study-specific OR and 95% CI. The area of the squares reflects the weight (inverse of the variance). The diamond represents the summary OR and 95% CI.

We carried out a meta-analysis of the hOGG1 Ser326Cys polymorphism overall, and in subgroups according to cancer types and ethnic groups under various genetic models (Table S2). Overall, we found that the hOGG1 Ser326Cys polymorphism was significantly associated with the risk of cancer (Cys/Cys vs. Ser/Ser: OR = 1.19, 95%CI = 1.09–1.30, *P*<0.001; Cys/Cys vs. Cys/Ser+Ser/Ser: OR = 1.16, 95%CI = 1.08–1.26, *P*<0.001; Table S2, Figure S2). In subgroup analyses by cancer types, we found that the hOGG1 Ser326Cys polymorphism was significantly associated with lung cancer (Cys/Cys vs. Ser/Ser: OR = 1.29, 95%CI = 1.16–1.44, *P*<0.001; Cys/Cys vs. Cys/ Ser+Ser/Ser: OR = 1.22, 95%CI = 1.12–1.33, *P*<0.001; Table S2, Figure 2), but not with colorectal, breast, bladder, prostate, and gastric cancer. In addition, we found that the hOGG1 Ser326Cys polymorphism was significantly associated with the risk of head and neck cancer (Cys/Cys vs. Ser/Ser: OR = 1.71, 95%CI = 1.05–2.78, *P* = 0.03).

In subgroup analyses by ethnicities, we found that the hOGG1 Ser326Cys polymorphism was associated with overall cancer risk in Asian population (Cys/Cys vs. Ser/Ser: OR = 1.21, 95%CI = 1.10–1.33, *P*<0.001; Cys/Cys vs. Cys/Ser+Ser/Ser: OR = 1.14, 95%CI = 1.03–1.26, *P* = 0.004; Cys/Cys+Cys/Ser vs. Ser/Ser: OR = 1.12, 95%CI = 1.05–1.19, *P*<0.001; Table S2). In subgroup analyses by age, we found that the hOGG1 Ser326Cys polymorphism was associated with overall cancer risk among cancer cases (<60 years) and cancer cases (≥60 years), respectively (Table S3). In addition, in subgroup analyses by sex, we found that the Ser326Cys polymorphism was not associated with overall cancer risk among women and men, respectively (Table S3).

### Ethnicity-specific effect of hOGG1 Ser326Cys polymorphism on cancer risk

When the data were analyzed in subgroups of subjects stratified by ethnicities, we found that the hOGG1 Ser326Cys polymorphism was significantly associated with overall cancer risk among Asians (Cys/Cys vs. Ser/Ser: OR = 1.21, 95%CI = 1.10–1.33, *P*<0.001; Table S2). The results of logistic regression analyses showed joint effects between Asians and hOGG1 Ser326Cys polymorphism (*P*<0.01).

### Test of heterogeneity

The heterogeneity was reckoned between each of the studies using the Q-test. Overall, the significant heterogeneity was found (Cys/Cys vs. Ser/Ser: *P*_heterogeneity_<0.001; Cys/ Ser vs. Ser/Ser: *P*_heterogeneity_<0.001; Cys/Cys vs. Cys/Ser+Ser/Ser: *P*_heterogeneity_<0.001; Cys/Cys+Cys/Ser vs. Ser/Ser: *P*_heterogeneity_<0.001). In stratified analyses by cancer types, we did not find the significant heterogeneity for lung cancer under two genetic models (Cys/Cys vs. Ser/Ser: *P*_heterogeneity_ = 0.40; Cys/Cys vs. Cys/ Ser+Ser/Ser: *P*_heterogeneity_ = 0.40).

### Sensitivity analysis

In the sensitivity analysis, the influence of each study on the pooled OR was examined by repeating the meta-analysis while omitting each study, one at a time. This procedure confirmed the stability of the overall result (data not shown). However, in the subgroup by ethnicities, sensitivity analyses show that P value of Z-test for statistical significance of the summary OR (Cys/Cys vs. Cys/Ser+Ser/Ser) among Caucasians is 0.06 when excluding one study by Obtulowicz et al.

### Publication bias

Begg's funnel plot and Egger's test were conducted to assess the publication bias of the literatures. The shape of funnel plots did not reveal any evidence of funnel plot asymmetry. Egger's test further provided statistical evidence of funnel plot symmetry (Cys/Cys vs. Ser/Ser: *P* = 0.28; Cys/Ser vs. Ser/Ser: *P* = 0.57; Cys/Cys vs. Cys/Ser+Ser/Ser: *P* = 0.20; Cys/Cys+Cys/Ser vs. Ser/Ser: *P* = 0.21). The results did not show any evidence of publication bias.

## Discussion

The hOGG1, which is generally involved in DNA repair, has been studied extensively on its relationship with different types of cancer, such as breast [6]–[18], prostate [19]–[25], pancreatic [26], [27], bladder [28]–[34], gallbladder [35]–[38], gastric [39]–[49], colorectal [50]–[63], esophageal [64]–[68], lung [69]–[85], cervical cancers [86], [87], and so on [88]–[101]. Previous conclusions of numerous studies on the association between the hOGG1 Ser326Cys polymorphism and cancer risk remain conflicting and contradictory. The conflicting results are possibly because of a small effect of the Ser326Cys polymorphism on cancer risk or the relatively low statistical power of published studies. Hence, this meta-analysis was needed to provide a quantitative approach for combining the different results.

The present meta-analysis, including 31,297 cancer cases and 39,033 controls, explored the relationship between the Ser326Cys polymorphism and overall cancer risk. In the meta-analysis, we found that the hOGG1 Ser326Cys polymorphism was significantly associated with overall cancer risk. In subgroup analyses by cancer types, the significant association between the hOGG1 Ser326Cys polymorphism and lung cancer risk was further detected. This result was consistent with previous study [109]. In addition, in subgroup analyses by ethnicities, we found that the hOGG1 Ser326Cys polymorphism was significantly associated with overall cancer risk in Asian population. However, sensitivity analyses suggested that the significant association between the Ser326Cys polymorphism and overall cancer risk among Caucasians lacked convincing evidence.

The hOGG1 encodes a DNA glycosylase that is thought to be involved in base excision repair of oxidatively damaged DNA [110]. The hOGG1 could catalyze the cleavage of the glycosylic bond between the modified base and the sugar moiety, leaving an abasic apurinic/apyrimidinic site in DNA; the resulting apurinic/apyrimidinic site is then incised, and the repair is completed by successive actions of a phosphodiesterase, a DNA polymerase, and a DNA liagse [111]–[113]. With respect to the important roles of hOGG1 in DNA repair, it is biologically plausible that hOGG1 Ser326Cys polymorphism may modulate the risk of cancer. This hypothesis was confirmed by our data. In addition, because of the relatively small sample size on head and neck cancer, the result about head and neck cancer needed further confirmation.

We did not find that hOGG1 Ser326Cys polymorphism was significantly associated with cancer risk in Caucasian population and other cancer types including breast, prostate, pancreatic, bladder, gallbladder, gastric, colorectal, and esophageal cancer, suggesting the influence of the genetic variant may be masked by the presence of other as-yet unidentified causal genes involved in carcinogenesis. In addition, we found that the frequency of the 326Cys allele was 47.07% among Asian controls, which was significantly higher than that of Caucasian controls (23.62%, *P*<0.001), which may also affect the roles of hOGG1 Ser326Cys polymorphism on cancer risk in Asians and Caucasians.

Several limitations of the meta-analysis should be addressed. First, limited data restricted our evaluation on potential gene-gene interaction. Second, there was not enough data on African population in this meta-analysis. Third, our results were based on unadjusted evaluation. In order to provide a more precise estimation on the basis of adjustment for confounders, well-designed studies are warranted by taking potential confounders such as alcohol and smoking into account.

In summary, this meta-analysis provided evidence of the association between hOGG1 Ser326Cys polymorphism and cancer risk, supporting the hypothesis that hOGG1 Ser326Cys polymorphism might be a low-penetrant susceptibility marker of lung cancer. Moreover, sophisticated gene-gene interaction should be considered in future analysis, which would lead a better, comprehensive understanding of the association between hOGG1 Ser326Cys polymorphism and cancer risk.

## Supporting Information

Figure S1 Frequencies of the variant alleles among controls stratified by ethnicities. The“○” and “*” represent outlier.
(TIF)

Figure S2 Forest plot of overall cancer risk associated with hOGG1 Ser326Cys polymorphism (for Cys/Cys vs. Ser/Ser). The squares and horizontal lines correspond to the study-specific OR and 95% CI. The area of the squares reflects the weight (inverse of the variance). The diamond represents the summary OR and 95% CI.
(TIF)

Table S1 Characteristics of studies included in the meta-analysis.
(DOC)

Table S2 Stratified analyses of the hOGG1 Ser326Cys polymorphism on cancer risk.
(DOC)

Table S3 Stratified analyses of the hOGG1 Ser326Cys polymorphism on cancer risk by age and sex.
(DOC)
