# Supplementary figures and images for: The Effect of hOGG1 Ser326Cys Polymorphism on Cancer Risk: Evidence from a Meta-Analysis
Source: PLoS One. 2011 Nov 17;6(11):e27545. doi: 10.1371/journal.pone.0027545 (PMC3219678; doi:10.1371/journal.pone.0027545)

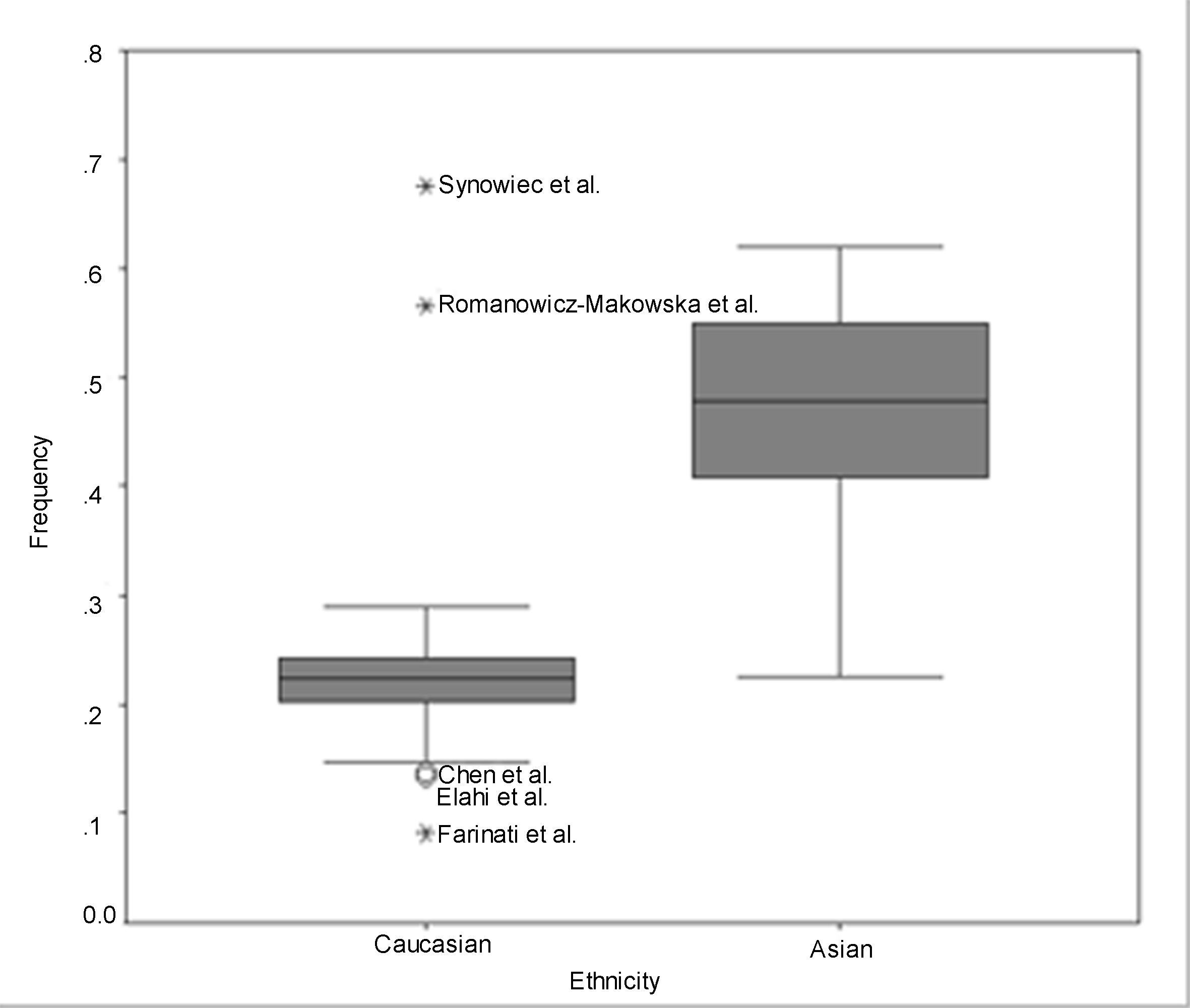

Supplement: Figure S1 — Frequencies of the variant alleles among controls stratified by ethnicities. The“○” and “*” represent outlier. (TIF) [file pone.0027545.s001.tif]

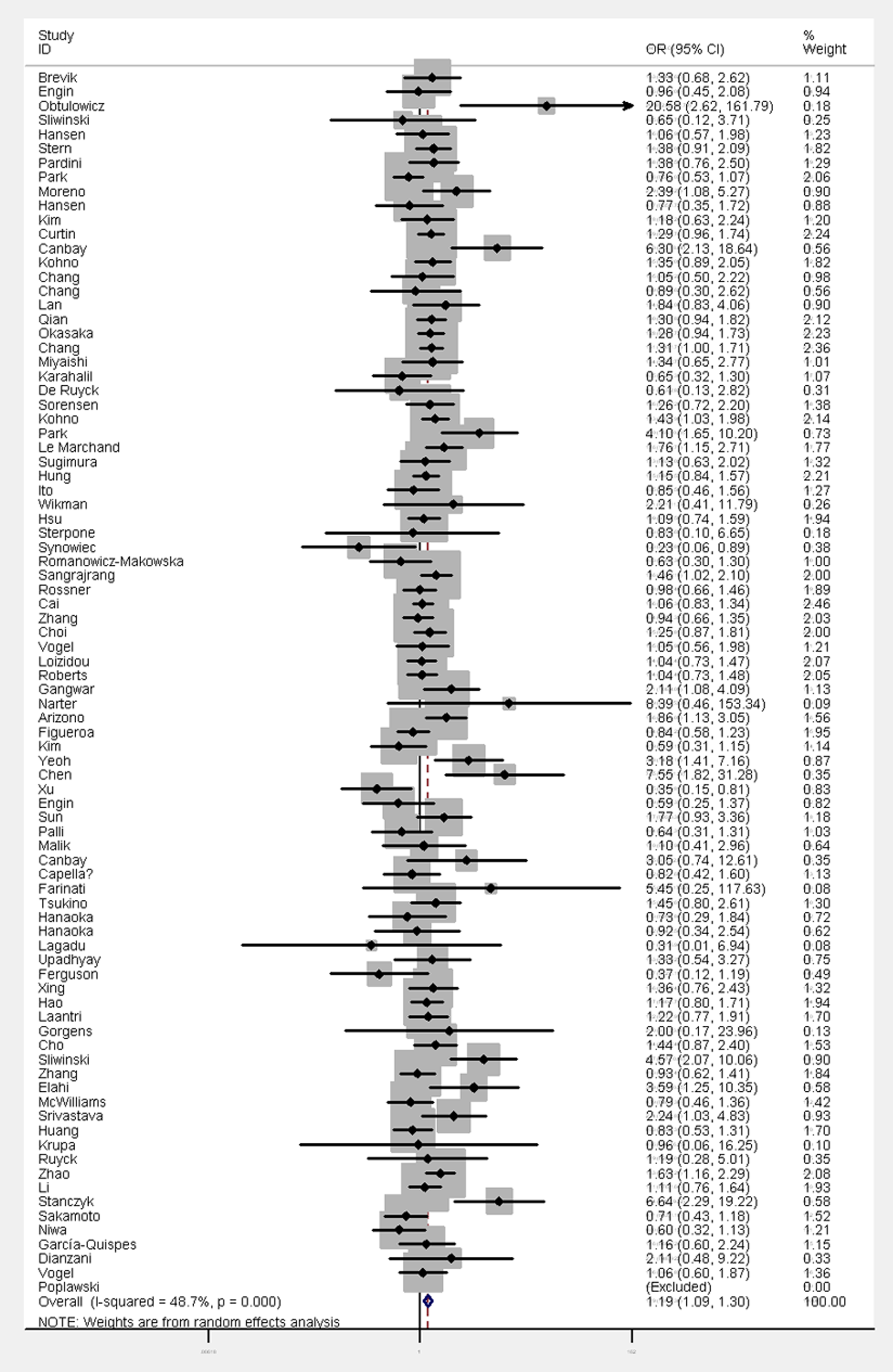

Supplement: Figure S2 — Forest plot of overall cancer risk associated with hOGG1 Ser326Cys polymorphism (for Cys/Cys vs. Ser/Ser). The squares and horizontal lines correspond to the study-specific OR and 95% CI. The area of the squares reflects the weight (inverse of the variance). The diamond represents the summary OR and 95% CI. (TIF) [file pone.0027545.s002.tif]
