# Supplementary material for: The Effect of hOGG1 Ser326Cys Polymorphism on Cancer Risk: Evidence from a Meta-Analysis
Source: PLoS One. 2011 Nov 17;6(11):e27545. doi: 10.1371/journal.pone.0027545 (PMC3219678; doi:10.1371/journal.pone.0027545)
Supplement: Table S1 — Characteristics of studies included in the meta-analysis. (DOC) [file pone.0027545.s003.doc]

Table S1. Characteristics of studies included in the meta-analysis.

| First author | | Year | Country | Ethnicity | Cases | | |  | Controls | | | | |
| --- | --- | --- | --- | --- | --- | --- | --- | --- | --- | --- | --- | --- | --- |
|  |  |  |  |  | Ser/Ser | Ser/Cys | Cys/Cys |  | Ser/Ser | Ser/Cys | Cys/Cys | P_HWE_ | Frequency  of 326Cys allele |
| Colorectal cancer | | |  |  |  |  |  |  |  |  |  |  |  |
| Brevik | | 2010 | USA | Mixed | 172 | 117 | 19 |  | 217 | 127 | 18 | 0.92 | 0.23 |
| Engin | | 2010 | Turkey | Mixed | 50 | 43 | 17 |  | 51 | 47 | 18 | 0.20 | 0.36 |
| Obtulowicz | | 2010 | Poland | Caucasian | 38 | 19 | 17 |  | 46 | 29 | 1 | 0.13 | 0.20 |
| Sliwinski | | 2009 | Poland | Caucasian | 52 | 46 | 2 |  | 68 | 28 | 4 | 0.61 | 0.18 |
| Hansen | | 2009 | Denmark | Caucasian | 220 | 137 | 16 |  | 467 | 277 | 32 | 0.25 | 0.22 |
| Kasahara | | 2008 | Japan | Asian | 17 | 51^a^ |  |  | 39 | 82 ^a^ |  | NA | NA |
| Stern | | 2007 | Singapore | Asian | 35 | 152 | 116 |  | 183 | 537 | 439 | 0.38 | 0.61 |
| Pardini | | 2008 | Czech | Caucasian | 336 | 168 | 28 |  | 331 | 181 | 20 | 0.44 | 0.21 |
| Park | | 2007 | Korea | Asian | 91 | 220 | 128 |  | 120 | 333 | 223 | 0.82 | 0.58 |
| Moreno | | 2006 | Spain | Caucasian | 225 | 114 | 23 |  | 210 | 104 | 9 | 0.36 | 0.19 |
| Hansen | | 2005 | Norway | Caucasian | 101 | 55 | 9 |  | 208 | 164 | 24 | 0.26 | 0.27 |
| Kim | | 2003 | Korea | Asian | 24 | 66 | 35 |  | 52 | 131 | 64 | 0.32 | 0.52 |
| Curtin | | 2009 | USA | Caucasian | 918 | 570 | 94 |  | 1172 | 686 | 93 | 0.56 | 0.22 |
| Canbay | | 2011 | Turkey | Caucasian | 31 | 40 | 8 |  | 171 | 69 | 7 | 0.99 | 0.17 |
| Lung cancer | |  |  |  |  |  |  |  |  |  |  |  |  |
| Kohno | | 2011 | Japan | Asian | 115 | 162 | 100 |  | 98 | 164 | 63 | 0.704 | 0.45 |
| Chang | | 2009 | USA | Mixed | 53 | 47 | 12 |  | 135 | 132 | 29 | 0.691 | 0.32 |
| Chang | | 2009 | USA | African | 170 | 78 | 6 |  | 202 | 70 | 8 | 0.521 | 0.15 |
| Lan | | 2004 | USA | Asian | 37 | 61 | 20 |  | 51 | 43 | 15 | 0.232 | 0.33 |
| Qian | | 2010 | China | Asian | 100 | 288 | 193 |  | 125 | 291 | 185 | 0.59 | 0.55 |
| Klinchid | | 2009 | Thailand | Asian | 7 | 69 ^a^ |  |  | 15 | 60 ^a^ |  | NA | NA |
| Okasaka | | 2009 | Japan | Asian | 117 | 257 | 141 |  | 250 | 544 | 236 | 0.07 | 0.49 |
| Chang | | 2009 | China | Asian | 142 | 518 | 436 |  | 154 | 482 | 361 | 0.74 | 0.60 |
| Miyaishi | | 2009 | Japan | Asian | 27 | 55 | 26 |  | 39 | 54 | 28 | 0.27 | 0.45 |
| Karahalil | | 2008 | Turkey | Mixed | 86 | 65 | 14 |  | 115 | 106 | 29 | 0.55 | 0.33 |
| De Ruyck | | 2007 | Belgium | Caucasian | 74 | 33 | 3 |  | 60 | 46 | 4 | 0.18 | 0.25 |
| Sorensen | | 2006 | Denmark | Caucasian | 254 | 155 | 22 |  | 479 | 284 | 33 | 0.26 | 0.22 |
| Kohno | | 2006 | Japan | Asian | 285 | 544 | 268 |  | 123 | 190 | 81 | 0.63 | 0.45 |
| Park | | 2004 | USA | Caucasian | 101 | 65 | 13 |  | 255 | 87 | 8 | 0.86 | 0.15 |
| Le Marchand | | 2002 | USA | Mixed | 123 | 110 | 65 |  | 177 | 175 | 53 | 0.35 | 0.35 |
| Sugimura | | 1999 | Japan | Asian | 85 | 115 | 41 |  | 63 | 107 | 27 | 0.08 | 0.41 |
| Hung | | 2005 | France | Caucasian | 1401 | 661 | 93 |  | 1368 | 716 | 79 | 0.22 | 0.20 |
| Ito | | 2002 | Japan | Asian | 40 | 71 | 27 |  | 68 | 118 | 54 | 0.84 | 0.47 |
| Wikman | | 2000 | Germany | Caucasian | 68 | 32 | 5 |  | 60 | 43 | 2 | 0.07 | 0.22 |
| Breast cancer | |  |  |  |  |  |  |  |  |  |  |  | . |
| Hsu | | 2010 | Taiwan | Asian | 64 | 165 | 172 |  | 87 | 231 | 215 | 0.06 | 0.62 |
| Sterpone | | 2010 | Italy | Caucasian | 18 | 23 | 2 |  | 15 | 14 | 2 | 0.59 | 0.29 |
| Synowiec | | 2008 | Poland | Caucasian | 10 | 19 | 12 |  | 4 | 23 | 21 | 0.51 | 0.68 |
| Romanowicz-  Makowska | | 2008 | Poland | Caucasian | 32 | 34 | 34 |  | 20 | 52 | 34 | 0.99 | 0.57 |
| Sangrajrang | | 2008 | Thailand | Asian | 112 | 232 | 162 |  | 104 | 217 | 103 | 0.63 | 0.50 |
| Rossner | | 2006 | USA | Caucasian | 615 | 375 | 51 |  | 653 | 385 | 55 | 0.86 | 0.23 |
| Cai | | 2006 | China | Asian | 186 | 534 | 382 |  | 214 | 537 | 416 | 0.08 | 0.59 |
| Zhang | | 2006 | USA | Caucasian | 967 | 532 | 72 |  | 760 | 424 | 60 | 0.93 | 0.22 |
| Choi | | 2003 | Korea | Asian | 105 | 227 | 134 |  | 111 | 244 | 113 | 0.36 | 0.50 |
| Vogel | | 2003 | Denmark | Caucasian | 256 | 147 | 22 |  | 245 | 169 | 20 | 0.18 | 0.24 |
| Loizidou | | 2009 | Cyprus | Mixed | 615 | 422 | 71 |  | 647 | 455 | 72 | 0.50 | 0.26 |
| Roberts | | 2011 | USA | Caucasian | 634 | 366 | 54 |  | 1125 | 670 | 92 | 0.543 | 0.23 |
| Bladder cancer | |  |  |  |  |  |  |  |  |  |  |  | . |
| Gangwar | | 2009 | Indian | Asian | 92 | 93 | 27 |  | 122 | 111 | 17 | 0.22 | 0.29 |
| Narter | | 2009 | Turkey | Mixed | 37 | 13 | 8 |  | 18 | 18 | 0 | 0.05 | 0.25 |
| Arizono | | 2008 | Japan | Asian | 61 | 107 | 83 |  | 67 | 135 | 49 | 0.20 | 0.46 |
| Huang | | 2007 | USA | Caucasian | 375 | 228 ^a^ |  |  | 348 | 260 ^a^ |  | NA | NA. |
| Figueroa | | 2007 | Spain | Caucasian | 649 | 383 | 56 |  | 596 | 361 | 61 | 0.52 | 0.24 |
| Kim | | 2005 | Korea | Asian | 37 | 90 | 26 |  | 38 | 70 | 45 | 0.30 | 0.52 |
| Prostate cancer | | |  |  |  |  |  |  |  |  |  |  |  |
| Zhang | | 2010 | USA | Mixed | 126 | 65 ^a^ |  |  | 118 | 78 ^a^ |  | NA | NA. |
| Yeoh | | 2009 | Australia | Caucasian | 38 | 57 | 21 |  | 69 | 50 | 12 | 0.50 | 0.28 |
| Chen | | 2003 | USA | Caucasian | 49 | 29 | 6 |  | 185 | 63 | 3 | 0.35 | 0.14 |
| Xu | | 2002 | USA | Caucasian | 182 | 106 | 10 |  | 96 | 63 | 15 | 0.32 | 0.27 |
| Gastric cancer | |  |  |  |  |  |  |  |  |  |  |  | . |
| Engin | | 2011 | Turkey | Mixed | 53 | 42 | 11 |  | 51 | 47 | 18 | 0.203 | 0.36 |
| Sun | | 2010 | China | Asian | 21 | 19 | 33 |  | 72 | 119 | 64 | 0.29 | 0.48 |
| Palli | | 2010 | Italy | Caucasian | 192 | 101 | 11 |  | 325 | 191 | 29 | 0.89 | 0.23 |
| Malik | | 2010 | India | Asian | 50 | 51 | 7 |  | 94 | 89 | 12 | 0.13 | 0.29 |
| Canbay | | 2010 | Turkey | Mixed | 24 | 13 | 3 |  | 171 | 69 | 7 | 0.99 | 0.17 |
| Capella´ | | 2008 | Spain | Caucasian | 156 | 76 | 11 |  | 688 | 391 | 59 | 0.72 | 0.22 |
| Farinati | | 2008 | Italy | Caucasian | 33 | 15 | 2 |  | 36 | 7 | 0 | 0.56 | 0.08 |
| Poplawski | | 2006 | Poland | Caucasian | 14 | 4 | 0 |  | 11 | 9 | 0 | 0.19 | 0.23 |
| Tsukino | | 2004 | Japan | Asian | 32 | 75 | 35 |  | 74 | 141 | 56 | 0.46 | 0.47 |
| Hanaoka | | 2001 | Japan | Asian | 20 | 29 | 9 |  | 44 | 56 | 27 | 0.25 | 0.43 |
| Hanaoka | | 2001 | Japan | Mixed | 133 | 67 | 8 |  | 123 | 74 | 8 | 0.44 | 0.22 |
| Esophageal cancer | | |  |  |  |  |  |  |  |  |  |  |  |
| Lagadu | | 2010 | France | Caucasian | 14 | 3 | 0 |  | 22 | 19 | 2 | 0.40 | 0.27 |
| Upadhyay | | 2010 | India | Asian | 59 | 66 | 10 |  | 94 | 89 | 12 | 0.13 | 0.29 |
| Ferguson | | 2008 | Ireland | Caucasian | 138 | 67 | 4 |  | 141 | 96 | 11 | 0.29 | 0.24 |
| Xing | | 2001 | China | Asian | 78 | 76 | 42 |  | 68 | 106 | 27 | 0.15 | 0.40 |
| Hao | | 2004 | China | Asian | 153 | 180 | 77 |  | 184 | 216 | 79 | 0.25 | 0.39 |
| Head and Neck cancer | | |  |  |  |  |  |  |  |  |  |  |  |
| Laantri | 2011 | | Morocco | African | 289 | 202 | 50 |  | 274 | 193 | 39 | 0.54 | 0.27 |
| Gorgens | 2007 | | Germany | Caucasian | 19 | 8 | 2 |  | 19 | 10 | 1 | 0.82 | 0.2 |
| Cho | | 2003 | Taiwan | Asian | 36 | 175 | 122 |  | 46 | 129 | 108 | 0.48 | 0.61 |
| Sliwinski | | 2011 | Poland | Caucasian | 109 | 128 | 28 |  | 160 | 111 | 9 | 0.05 | 0.23 |
| Zhang | | 2004 | USA | Caucasian | 447 | 220 | 39 |  | 739 | 388 | 69 | 0.06 | 0.22 |
| Elahi | | 2002 | USA | Caucasian | 104 | 54 | 9 |  | 249 | 76 | 6 | 0.94 | 0.13 |
| Gallbladder cancer | | |  |  |  |  |  |  |  |  |  |  |  |
| Srivastava | | 2010 | India | Asian | 117 | 92 | 21 |  | 137 | 82 | 11 | 0.78 | 0.23 |
| Huang | | 2008 | China | Asian | 96 | 107 | 33 |  | 268 | 355 | 111 | 0.71 | 0.39 |
| ALL | |  |  |  |  |  |  |  |  |  |  |  |  |
| Li | | 2011 | China | Asian | 69 | 174 | 172 |  | 79 | 255 | 177 | 0.41 | 0.60 |
| Stanczyk | | 2011 | Poland | Caucasian | 43 | 37 | 17 |  | 84 | 42 | 5 | 0.93 | 0.20 |
| Cervical cancer | |  |  |  |  |  |  |  |  |  |  |  |  |
| Niwa | | 2005 | Japan | Asian | 37 | 60 | 19 |  | 94 | 146 | 80 | 0.13 | 0.48 |
| Farkasova | | 2008 | Slovak | Caucasian | 10 | 8^a^ |  |  | 10 | 15^a^ |  | NA | NA |
| Mixed cancer | |  |  |  |  |  |  |  |  |  |  |  |  |
| García-Quispes | | 2011 | Spain | Caucasian | 253 | 125 | 19 |  | 294 | 154 | 19 | 0.84 | 0.21 |
| Dianzani | | 2006 | Italy | Caucasian | 53 | 23 | 5 |  | 67 | 40 | 3 | 0.30 | 0.21 |
| Vogel | | 2004 | Denmark | Caucasian | 169 | 121 | 29 |  | 167 | 125 | 27 | 0.60 | 0.28 |
| McWilliams | | 2008 | USA | Caucasian | 268 | 178 | 23 |  | 339 | 223 | 37 | 0.97 | 0.25 |
| Krupa | | 2011 | Poland | Caucasian | 23 | 6 | 1 |  | 22 | 7 | 1 | 0.64 | 0.15 |
| Ruyck | | 2004 | Belgium | Caucasian | 39 | 20 | 3 |  | 93 | 51 | 6 | 0.76 | 0.21 |
| Zhao | | 2010 | China | Asian | 85 | 257 | 230 |  | 112 | 277 | 186 | 0.63 | 0.56 |
| Sakamoto | | 2006 | Japan | Asian | 56 | 110 | 43 |  | 73 | 123 | 79 | 0.08 | 0.51 |

NA, not available; a, genotypes as “Ser/Cys + Cys/Cys”.
