# Supplementary material for: The Effect of hOGG1 Ser326Cys Polymorphism on Cancer Risk: Evidence from a Meta-Analysis
Source: PLoS One. 2011 Nov 17;6(11):e27545. doi: 10.1371/journal.pone.0027545 (PMC3219678; doi:10.1371/journal.pone.0027545)
Supplement: Table S2 — Stratified analyses of the hOGG1 Ser326Cys polymorphism on cancer risk. (DOC) [file pone.0027545.s004.doc]

Table S2 Stratified analyses of the hOGG1 Ser326Cys polymorphism on cancer risk.

| Vaviables | N^a^ | Cases/  Controls |  | Cys/Cys vs. Ser/Ser | | |  | Cys/Ser vs. Ser/Ser | | |  | Cys/Cys + Cys/Ser  vs. Ser/Ser | | |  | Cys/Cys vs.  Cys/Ser+Ser/Ser | | |
| --- | --- | --- | --- | --- | --- | --- | --- | --- | --- | --- | --- | --- | --- | --- | --- | --- | --- | --- |
|  |  |  |  | OR(95% CI) | *P* | *P*^b^ |  | OR(95% CI) | *P* | *P*^b^ |  | OR(95% CI) | *P* | *P*^b^ |  | OR(95% CI) | *P* | *P*^b^ |
| Total | 91 | 31297/  39033 |  | 1.19(1.09-1.30) | <0.001 | <0.001 |  | 1.03(0.97-1.08) | 0.35 | <0.001 |  | 1.06(1.00-1.12) | 0.03 | <0.001 |  | 1.16(1.08-1.26) | <0.001 | <0.001 |
| Colorectal cancer | 14 | 4620/  7082 |  | 1.31(1.00-1.72) | 0.06 | 0.004 |  | 1.11(0.94-1.32) | 0.21 | 0.001 |  | 1.16(0.99-1.36) | 0.07 | 0.001 |  | 1.20(0.96-1.50) | 0.11 | 0.02 |
| Lung  cancer | 19 | 8156/  8844 |  | 1.29(1.16-1.44) | <0.001 | 0.40 |  | 1.04(0.93-1.17) | 0.49 | 0.01 |  | 1.01(0.98-1.24) | 0.096 | 0.001 |  | 1.22(1.12-1.33) | <0.001 | 0.40 |
| Breast cancer | 12 | 7858/  8609 |  | 1.06(0.95-1.19) | 0.31 | 0.42 |  | 0.98(0.92-1.05) | 0.59 | 0.33 |  | 0.99(0.93-1.06) | 0.86 | 0.35 |  | 1.07(0.97-1.17) | 0.17 | 0.46 |
| Bladder cancer | 6 | 2365/  2316 |  | 1.26(0.72-2.21) | 0.42 | 0.01 |  | 0.97(0.84-1.13) | 0.73 | 0.15 |  | 0.95(0.84-1.07) | 0.39 | 0.30 |  | 1.26(0.66-2.42) | 0.48 | <0.001 |
| Prostate cancer | 4 | 689/  752 |  | 1.92(0.32-11.42) | 0.47 | <0.001 |  | 1.43(0.83-2.48) | 0.20 | 0.03 |  | 1.27(0.72-2.21) | 0.41 | <0.001 |  | 1.60(0.34-7.48) | 0.55 | <0.001 |
| Gastric cancer | 11 | 1350/  3162 |  | 1.04(0.81-1.35) | 0.75 | 0.23 |  | 0.93(0.80-1.08) | 0.32 | 0.37 |  | 0.95(0.82-1.09) | 0.43 | 0.33 |  | 1.10(0.77-1.56) | 0.61 | 0.05 |
| Esophageal cancer | 5 | 967/  1166 |  | 1.12(0.84-1.49) | 0.45 | 0.32 |  | 0.81(0.60-1.10) | 0.18 | 0.06 |  | 0.85(0.63-1.15) | 0.30 | 0.05 |  | 1.21(0.93-1.58) | 0.15 | 0.24 |
| Head and Neck cancer | 6 | 2041/  2626 |  | 1.71(1.05-2.78) | 0.03 | 0.007 |  | 1.27(0.97-1.66) | 0.09 | 0.01 |  | 1.32(0.99-1.77) | 0.06 | 0.001 |  | 1.41(0.93-2.12) | 0.11 | 0.01 |
| Gallbladder cancer | 2 | 466/  964 |  | 1.29(0.49-3.40) | 0.60 | 0.03 |  | 1.04(0.67-1.60) | 0.87 | 0.08 |  | 1.08(0.64-1.81) | 0.77 | 0.03 |  | 1.27(0.59-2.70) | 0.54 | 0.07 |
| ALL | 2 | 512/  642 |  | 2.53(0.44-14.50) | 0.30 | 0.002 |  | 1.12(0.52-2.43) | 0.77 | 0.02 |  | 1.40(0.58-3.36) | 0.45 | 0.01 |  | 2.44(0.63-9.38) | 0.20 | 0.01 |
| Ethnicity |  |  |  |  |  |  |  |  |  |  |  |  |  |  |  |  |  |  |
| Caucasian | 42 | 16113/  20408 |  | 1.20(0.99-1.44) | 0.06 | <0.001 |  | 1.00(0.92-1.08) | 0.97 | <0.001 |  | 1.02(0.94-1.11) | 0.64 | <0.001 |  | 1.19(1.00-1.39) | 0.04^c^ | <0.001 |
| Asian | 35 | 11606/  14189 |  | 1.21(1.10-1.33) | <0.001 | 0.06 |  | 1.07(1.00-1.14) | 0.05 | 0.20 |  | 1.12(1.05-1.19) | <0.001 | 0.42 |  | 1.14(1.03-1.26) | 0.004 | <0.001 |

a number of included studies.

b *P* value of Q-test for heterogeneity test.

c Sentivity analyses show that *P* value of Z-test for statistical significance of the summary OR is 0.06 when excluding one study by Obtulowicz et al.
