# Supplementary material for: The Effect of hOGG1 Ser326Cys Polymorphism on Cancer Risk: Evidence from a Meta-Analysis
Source: PLoS One. 2011 Nov 17;6(11):e27545. doi: 10.1371/journal.pone.0027545 (PMC3219678; doi:10.1371/journal.pone.0027545)
Supplement: Table S3 — Stratified analyses of the hOGG1 Ser326Cys polymorphism on cancer risk by age and sex. (DOC) [file pone.0027545.s005.doc]

Table S3. Stratified analyses of the hOGG1 Ser326Cys polymorphism on cancer risk by age and sex.

| Vaviables | N^a^ | Cases/  Controls |  | Cys/Cys vs. Ser/Ser | | |  | Cys/Ser vs. Ser/Ser | | |  | Cys/Cys + Cys/Ser  vs. Ser/Ser | | |  | Cys/Cys vs.  Cys/Ser+Ser/Ser | | |
| --- | --- | --- | --- | --- | --- | --- | --- | --- | --- | --- | --- | --- | --- | --- | --- | --- | --- | --- |
|  |  |  |  | OR(95% CI) | *P* | *P*^b^ |  | OR(95% CI) | *P* | *P*^b^ |  | OR(95% CI) | *P* | *P*^b^ |  | OR(95% CI) | *P* | *P*^b^ |
| Mean age |  |  |  |  |  |  |  |  |  |  |  |  |  |  |  |  |  |  |
| ＜60 years | 17 | 5885/  7196 |  | 1.16(1.03-1.32) | 0.02 | 0.11 |  | 0.98(0.91-1.07) | 0.70 | 0.30 |  | 1.02(0.94-1.10) | 0.62 | 0.10 |  | 1.13(1.02-1.25) | 0.02 | 0.13 |
| ≥60 years | 25 | 5158/  6930 |  | 1.49(1.13-1.97) | 0.01 | <0.001 |  | 1.10(0.92-1.31) | 0.29 | <0.001 |  | 1.13(0.96-1.32) | 0.15 | <0.001 |  | 1.38(1.07-1.78) | 0.01 | <0.001 |
| Sex |  |  |  |  |  |  |  |  |  |  |  |  |  |  |  |  |  |  |
| Female | 15 | 8068/  9029 |  | 1.04(0.93-1.17) | 0.49 | 0.28 |  | 0.98(0.92-1.05) | 0.61 | 0.41 |  | 0.99(0.92-1.07) | 0.87 | 0.27 |  | 1.05(0.96-1.15) | 0.32 | 0.23 |
| Male | 4 | 689/  752 |  | 1.92(0.32-11.42) | 0.47 | <0.001 |  | 1.43(0.83-2.48) | 0.20 | 0.03 |  | 1.27(0.72-2.21) | 0.41 | <0.001 |  | 1.60(0.34-7.48) | 0.55 | <0.001 |

a number of included studies.

b *P* value of Q-test for heterogeneity test.
